# Relevance of choroid plexus volumes in multiple sclerosis

**DOI:** 10.1186/s12987-025-00656-7

**Published:** 2025-05-08

**Authors:** Britta Krieger, Barbara Bellenberg, Anna Katharina Roenneke, Ruth Schneider, Theodoros Ladopoulos, Zainab Abbas, Rebekka Rust, Tanja Schmitz-Hübsch, Claudia Chien, Ralf Gold, Friedemann Paul, Carsten Lukas

**Affiliations:** 1 Institute of Neuroradiology, St. Josef Hospital, Ruhr University Bochum, Gudrunstr. 56, 44791 Bochum, Germany; 2 Department of Neurology, St. Josef Hospital, Ruhr University Bochum, 44791 Bochum, Germany; 3 Experimental and Clinical Research Center, Max Delbrueck Center for Molecular Medicine, Charité-University Hospital Berlin, 10117 Berlin, Germany; 4 Experimental and Clinical Research Center, NeuroCure Clinical Research Center, Department of Psychiatry and Neurosciences, Charité-University Hospital Berlin, 10117 Berlin, Germany

**Keywords:** Multiple sclerosis, MRI, Neuroinflammation, Choroid plexus

## Abstract

**Background:**

The choroid plexus (ChP) plays a pivotal role in inflammatory processes that occur in multiple sclerosis (MS). The enlargement of the ChP in relapsing-remitting multiple sclerosis (RRMS) is considered to be an indication of disease activity and has been associated with periventricular remyelination failure. This cross-sectional study aimed to identify the relationship between ChP and periventricular tissue damage which occurs in MS, and to elucidate the role of neuroinflammation in primary progressive multiple sclerosis (PPMS).

**Methods:**

ChP volume was assessed by a novel deep learning segmentation method based on structural MRI data acquired from two centers. In total, 141 RRMS and 64 PPMS patients were included, along with 75 healthy control subjects. In addition, T1w/FLAIR ratios were calculated within periventricular bands to quantify microstructural tissue damage and to assess its relationship to ChP volume.

**Results:**

When compared to healthy controls, ChP volumes were significantly increased in RRMS, but not in patients with PPMS. T1w/FLAIR ratios in the normal appearing white matter (NAWM) showing periventricular gradients were decreased in patients with multiple sclerosis when compared to healthy control subjects and lower T1w/FLAIR ratios radiating out from the lateral ventricles were found in patients with PPMS. A relationship between ChP volume and T1w/FLAIR ratio in NAWM was found within the inner periventricular bands in RRMS patients. A longer duration of disease was associated with larger ChP volumes only in RRMS patients. Enlarged ChP volumes were also significantly associated with reduced cortex volumes and increased lesion volumes in RRMS.

**Conclusions:**

Our analysis confirmed that the ChP was significantly enlarged in patients with RRMS, which was related to brain lesion volumes and which suggested a dynamic development as it was associated with disease duration. Plexus enlargement was further associated with periventricular demyelination or tissue damage assessed by T1w/FLAIR ratios in RRMS. Furthermore, we did not find an enlargement of the ChP in patients with PPMS, possibly indicating the reduced involvement of inflammatory processes in the progressive phase of MS. The association between enlarged ChP volumes and cortical atrophy in RRMS highlighted the vulnerability of structures close to the CSF.

**Supplementary Information:**

The online version contains supplementary material available at 10.1186/s12987-025-00656-7.

## Background

The choroid plexus (ChP) plays a pivotal role for the immune response within the CNS as it is responsible for both the production of CSF and the regulation of immune cells and other molecules that enter the brain through the meningeal blood vessels and the pial layer under inflammatory conditions [1]. Thus, the ChP is important for immunological functionality as it represents a key component of the blood-cerebrospinal fluid barrier (BCSFB), one of the main barriers for the transport of solutes into the CNS. Proposed as a surrogate marker for immune cell migration into the CNS, the ChP is a crucial structure to study relating to acute inflammatory processes in neurological diseases such as multiple sclerosis (MS) [2, 3]. In MS, pro-inflammatory immune cells entering the CNS from peripheral regions can trigger disease-specific inflammatory cascades [4].

Recent studies detected associations between ChP volume and the degree of neuroinflammation, thus highlighting that this structure can be a marker for MS disease severity and activity [5–8]. To further investigate the role of ChP in disease activity in patients with MS, we compared ChP volumes between relapsing-remitting multiple sclerosis (RRMS) and primary progressive multiple sclerosis (PPMS) patients and healthy control subjects. We also aimed to confirm ChP enlargement in RRMS. Since PPMS is less inflammation-driven, we hypothesized that ChP enlargement in PPMS is less or not observable, when compared to RRMS. A distinct involvement when compared to RRMS would further help us to differentiate between disease types.

To perform a more in-depth analysis of ChP volume changes and their potential effects on the surrounding brain tissue, we further made use of a recently suggested biomarker for quantifying microstructural tissue damage. Cappelle et al. [9] recently showed that the ratios of T1-weighted (T1w) to fluid-attenuated inversion recovery (FLAIR) images appear to be a feasible option for identifying changes in tissue integrity representing myelin content, axonal degradation, or dendrite density in the brain. In the present study, we calculated T1w/FLAIR ratios evaluated within concentric bands of the non-lesional normal appearing white matter (NAWM) starting at the lateral ventricular margin and extending towards the brain parenchyma. Using ChP volumes and T1w/FLAIR ratios, we aim to evaluate and compare different phases of MS which may show marked changes in neuroinflammation and cerebral tissue damage.

## Methods

### Study participants

This study included subjects examined at University Hospital Bochum and at Charité Berlin. The participants from University Hospital Bochum received MRI in the context of clinical routine examinations between September 2018 and June 2022 and were retrospectively reviewed in a cross-sectional manner. MS patients were included if they fulfilled the McDonald 2017 criteria [10]. Healthy control subjects (CS) with no neurological deficits were scanned during clinical routine MRI examinations to exclude intracranial pathology. Subjects were excluded if they exhibited any forms of intracranial pathology, including small vessel disease, ischemic or haemorrhagic stroke, hydrocephalus, or tumours, or if MRI images were of insufficient quality.

Patients and healthy controls from Charité Berlin were examined between May 2009 and February 2016 in the context of the SUPREMES study [11]. Patients were eligible if they fulfilled the 2005 revised McDonald criteria for MS, had been diagnosed of RRMS or PPMS, were aged between 18 and 65 years, had an expanded disability status scale (EDSS) score of 3 to 8 at screening, and had a relapse-free period of at least 30 days prior to randomization. Patients were excluded if they had been diagnosed with a major systemic or CNS disease, especially Parkinson’s, Huntington’s, or Alzheimer’s disease, or if they exhibited clinically relevant pre-defined laboratory abnormalities (e.g., aminotransferases that were > 3.5-fold higher than the normal limit).

### Magnetic resonance imaging

In Bochum, MRI acquisition was performed on a 1.5 Tesla Siemens Aera scanner with a 16-channel head/neck matrix coil. The standardized MRI protocols for MS included sagittal 3D T1-weighted (T1w) MPRAGE (repetition time: 10 ms; echo time: 4.6 ms; inversion time: 1000 ms; voxel size: 1 × 1 × 1 mm^3^; acquisition matrix: 240 × 240, 180 slices) and sagittal 3D fluid-attenuated inversion recovery (FLAIR) scans (repetition time: 5000 ms; echo time: 332 ms; inversion time: 1800 ms; flip angle: 120°; number of excitations: 1; voxel size: 1 × 1 × 1 mm^3^; acquisition matrix: 256 × 230, 160 slices).

In Berlin, MRI data were generated on a 1.5 Tesla Siemens Sonata scanner with a 16-channel coil. The standardized protocols included 3D T1w MPRAGE (repetition time: 2110 ms; echo time: 4.38 ms; inversion time: 1100 ms; voxel size: 0.5 × 0.5 × 1 mm^3^; acquisition matrix: 352 × 512, 160 slices) and 2D FLAIR scans (repetition time: 10000 ms, echo time: 108 ms, inversion time: 2500 ms, flip angle: 150°, number of excitations: 1, voxel size: 0.5 × 0.5 × 3 mm^3^; acquisition matrix: 512 × 512, 44 slices).

### Image processing

T1w images were segmented by Sequence Adaptive Multimodal SEGmentation (SAMSEG) in FreeSurfer [12]. FLAIR images were registered to corresponding T1w images by the linear registration program “mri_coreg” in Freesurfer and were included in the SAMSEG pipeline to gain a better segmentation result and to be able to use the dedicated option for lesion segmentation [13]. Analyses were conducted by FreeSurfer Version 7.3 [14]. To ensure the same voxel sizes for all segmentations, T1w data from Berlin were resampled to 1 × 1 × 1 mm^3^ prior to processing using FMRIB Software Library (FSL) [15]. For ChP segmentation, we made use of a recently developed deep learning segmentation method showing improved performance compared to FreeSurfer [16]. Examples for ChP segmentation were visualized in the supplement (Supplement Fig. 1). To reduce intra-individual variability, ChP, cortical gray matter (GM), and white matter (WM) volumes were normalized to intracranial volume.

### Creation of T1w/FLAIR ratio images

The registered T1w and FLAIR images from SAMSEG were further processed based on a method described by Cappelle et al. [9] to acquire T1w/FLAIR ratios. These authors demonstrated the feasibility of using T1w/FLAIR ratios as a biomarker for tissue integrity in MS and compared different methods for image calibration to account for between-subject and within-subject intensity variations. The authors concluded that non-linear image calibration methods produced the best results; therefore, we chose their nonlinear histogram calibration with subject template by utilizing our own study templates from T1w and FLAIR images. The impact of lesion voxels on ratios were accounted for by excluding lesions obtained by SAMSEG from the brain tissue mask that was used for calibration. Examples for T1/FLAIR ratio images of both centers and their corresponding raw T1w and FLAIR images as well as calibrated images were visualized in the supplement (Supplement Fig. 2).

### Periventricular bands

The ventricle masks derived from SAMSEG were used to create ten periventricular bands in NAWM. The supratentorial ventricle mask was repeatedly dilated by one-voxel (1 × 1 × 1 mm^3^) for each band using the DilM option in the ‘fslmaths’ tool in FSL (FMRIB software library) [15] and subtracted from the previous band mask to acquire concentric periventricular one-voxel thick bands representing NAWM layers located 1 to 10 mm from the ventricles (Suppl. Figure 1). The band masks were further multiplied by the brain WM mask to exclude lesions and the cortex. The first band adjacent to the lateral ventricles was excluded from these analyses due to the influence of CSF-white matter partial volume effects on ventricle borders. T1w/FLAIR ratios were calculated within each periventricular band using the ‘fslstats’ tool in FSL. Linear regression analysis was then performed to investigate the relationship between T1w/FLAIR ratios and ChP volumes.

### Clinical parameters

Disease duration and EDSS data were acquired from the electronic health record system. EDSS was determined by experienced neurologists to assess clinical disability in MS based on several functional systems.

### Statistical analysis

Statistical analysis was performed with R software (version 4.0.1) [17]. Bilateral ChP volumes were calculated by summing both hemispheres. Comparisons of ChP between the CS, RRMS, and PPMS groups were performed by analyses of covariance (ANCOVA) with pairwise post-hoc tests, using age, sex, and center as covariates and *p* value adjustment equivalent to the Tukey test. Demographics were compared between centers using two sample t-tests. Associations between ChP and disease duration, EDSS, cortical GM volumes, WM volumes, lesion volumes, lateral ventricle volume (LVV), and T1w/FLAIR ratios were assessed using multivariable linear regression analysis with age and sex as covariates. Within each periventricular band, the relationship between ChP volumes and T1w/FLAIR ratios were analysed using multivariable linear regression analysis with age, sex and center as covariates and multiple comparison correction using Benjamini-Hochberg procedure.

### Data availability

Raw MRI and clinical data cannot be made available due to data protection regulations. Other data can be shared on reasonable request to the corresponding author.

## Results

### Study sample

Initially, we retrieved 315 MS patients and CS. However, 35 were excluded due to incomplete data sets or insufficient image quality, e.g. artefacts or cropped images, resulting in 75 CS, 64 PPMS and 141 RRMS patients. The demographical data of the final cohort is summarized in Table 1. Overall, PPMS patients had higher EDSS scores (*p* < 0.001, 95% CI -3.89,-2.88) and longer disease durations (*p* = 0.01, 95% CI -4.87,-0.59) when compared to RRMS patients. Furthermore, PPMS patients were older than the CS and RRMS patients (*p* < 0.01, 95% CI 16.0,24.8 and − 19.8,-12.0 respectively). The gender distribution was not significantly different between groups. PPMS patients from Bochum showed longer disease durations (mean 12 years) than Berlin (mean 7 years) (*p* < 0.001, 95% CI -7.9,-2.5) and higher ages (mean 60 vs. 52 years) (*p* < 0.001, 95% CI -12,-4).

**Table 1:** Demographic data

	CS *N* = 75^*1*^	PPMS *N* = 64^*1*^	RRMS *N* = 141^*1*^
center			
Berlin	25 (33%)	22 (34%)	22 (16%)
Bochum	50 (67%)	42 (66%)	119 (84%)
age [years]	34 (28, 43)	56 (52, 62)	40 (30, 48)
sex			
male	26 (35%)	31 (48%)	49 (35%)
female	49 (65%)	33 (52%)	92 (65%)
disease duration [years]		9 (7, 14)	7 (2, 13)
Unknown		0	3
EDSS		6.00 (4.75, 6.50)	2.00 (1.50, 3.00)
Unknown		1	12

^*1*^ n (%); Median (IQR)

CS: control subjects, PPMS: primary progressive multiple sclerosis, RRMS: relapsing-remiting multiple sclerosis, EDSS: expanded disability status scale, IQR: inter quartile range

### Choroid plexus enlargement in multiple sclerosis patients

**Fig. 1:**
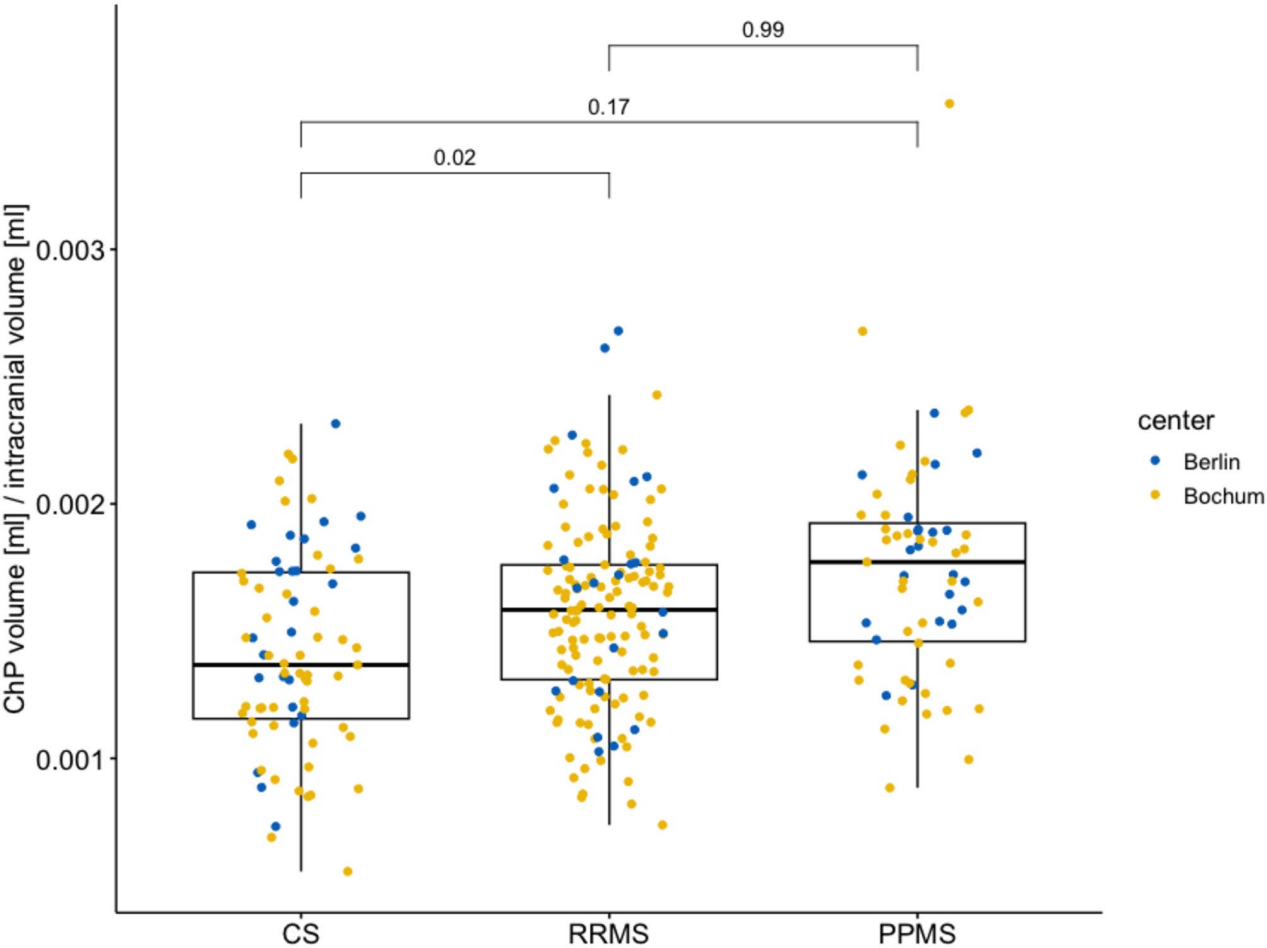
**Choroid Plexus volumes in control subjects, relapsing-remitting, and primary progressive multiple sclerosis**. Comparison of choroid plexus (ChP) volumes normalized for intracranial volumes between control subjects (CS) and patients with relapsing-remitting (RRMS) and primary progressive multiple sclerosis (PPMS). Values from the two centers are coloured in blue (Berlin) and yellow (Bochum). *P*-values were obtained from pairwise Tukey post-hoc tests following the analysis of covariance with age, sex, and center as covariates

Our analysis showed that ChP was increased in RRMS and in PPMS when compared to the CS group, but did not reach significance in PPMS after adjustment for age, sex and center (Fig. 1). Estimated marginal means resulting from the model were visualized in Supplement Fig. 4.

**Fig. 2:**
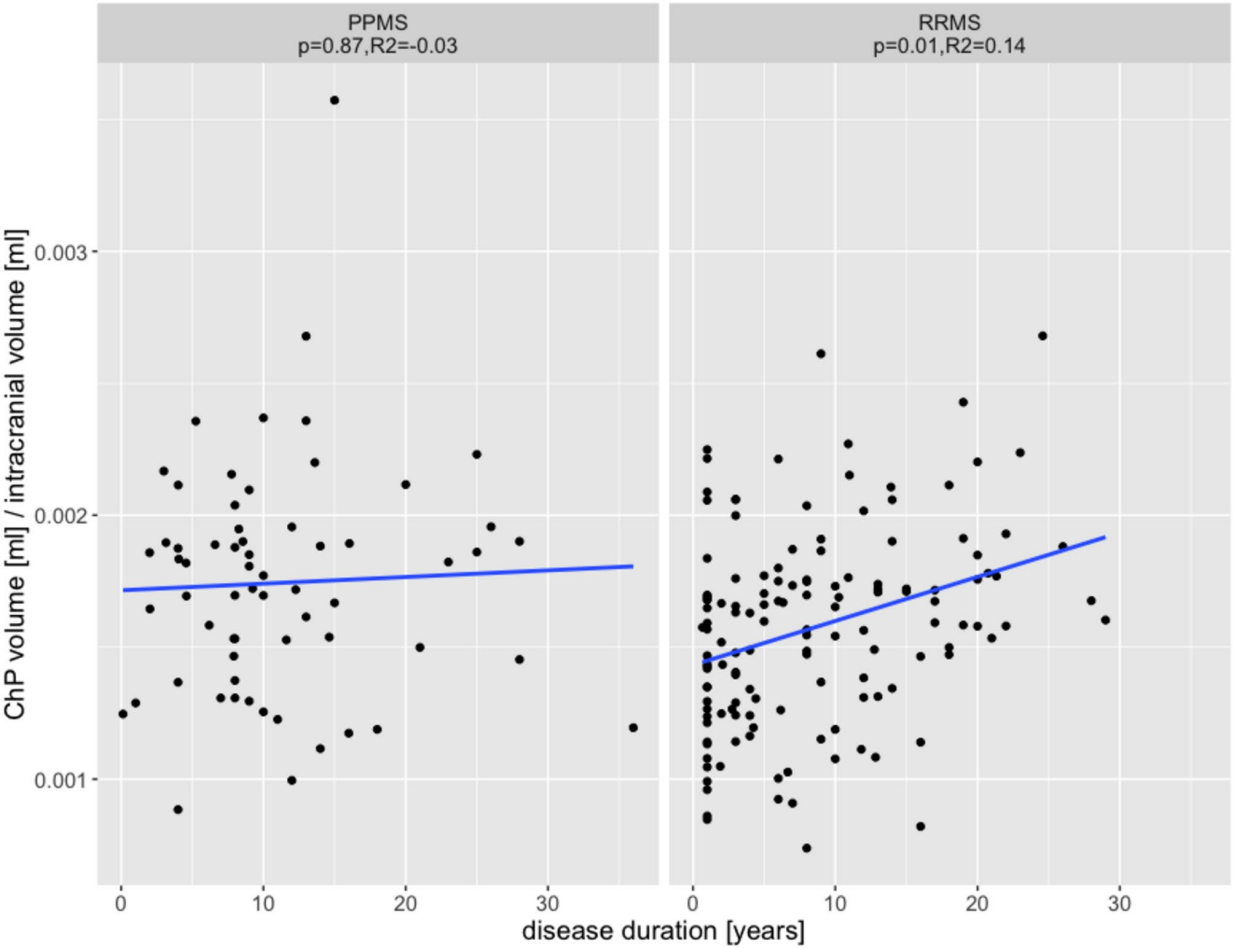
**Relationship between choroid plexus volumes and disease duration**. Relationship between choroid plexus (ChP) volumes normalized for intracranial volume and disease duration for patients with relapsing-remitting (RRMS) and primary progressive multiple sclerosis (PPMS). Blue lines represent fitted linear regression lines. *P*-values (for ChP vs. disease duration) and adjusted R2 were obtained from linear regression analyses with age and sex as covariates

A positive association between disease duration and ChP was observed for RRMS patients (*p* = 0.02, 95% CI 1.3 × 10^− 6^,1.9 × 10^− 5^), but not for PPMS patients (*p* = 0.86, 95% CI -1.7 × 10^− 5^,2.0 × 10^− 5^) (Fig. 2). Moreover, with a longer disease duration, the ChP of RRMS patients increased to become similar to that of PPMS patients. No significant relationships between ChP and EDSS were detected for RRMS and PPMS.

**Fig. 3:**
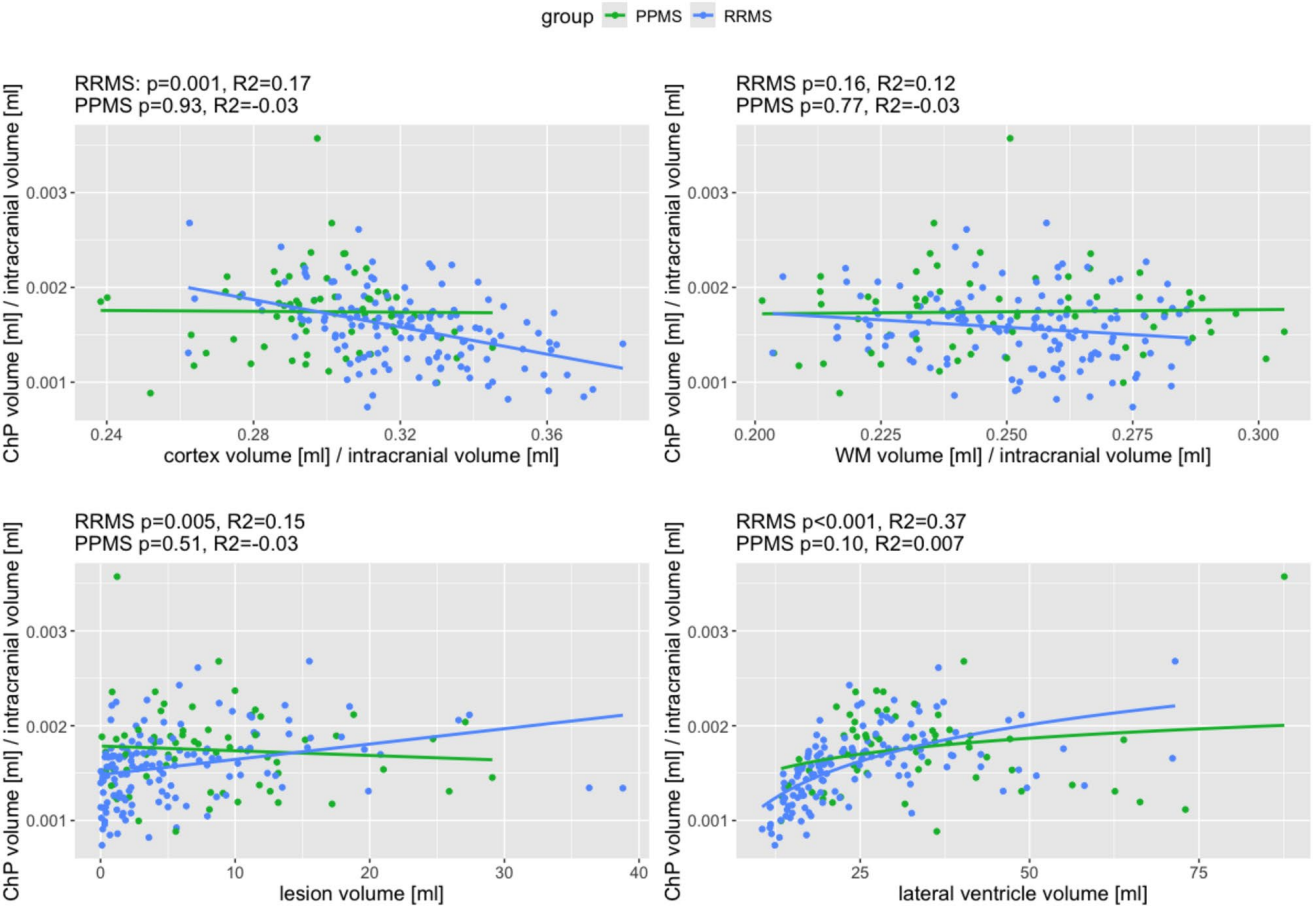
**Relationship between choroid plexus volumes and brain volumes**. Relationship between choroid plexus (ChP) volumes normalized to intracranial volume and cortical gray matter volume, white matter volume, lesion volume, or lateral ventricle volume for patients with relapsing-remitting (RRMS) and primary progressive multiple sclerosis (PPMS). *P*-values (for ChP vs. volumes) and adjusted R2 were obtained from linear (or non-linear for lateral ventricle volume) regression analyses with age and sex as covariates

Analyses showed that ChP was significantly associated with total brain lesion volume for patients with RRMS (*p* = 0.005, 95% CI 3.7 × 10^− 6^,2.1 × 10^− 5^), but not in PPMS (*p* = 0.51, 95% CI -2.2 × 10^− 5^,1.1 × 10^− 5^) (Fig. 3). Furthermore, decreased cortical GM volumes were significantly related to higher ChP volumes in RRMS (*p* = 0.001, 95% CI -0.009,-0.002), but not in PPMS (*p* = 0.93, 95% CI -0.005,0.006). No association between ChP and WM volumes was found. In RRMS, higher ChP volumes were significantly associated with LVV (*p* < 0.001, 95% CI 3.8 × 10^− 4^,6.6 × 10^− 4^) using a logarithmic function which fitted the values better than linear function (R^2^ = 0.37 vs. R^2^ = 0.27). Therefore, the association seemed to be driven by values of LVV below 20 ml.

### T1w/FLAIR ratios in the periventricular bands of normal-appearing white matter

**Fig. 4:**
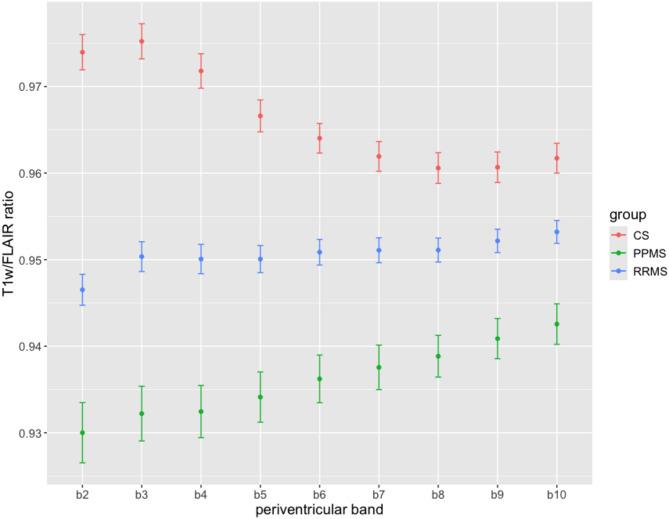
**T1w/FLAIR ratios within periventricular bands**. Mean T1w/FLAIR ratios of normal-appearing white matter in each periventricular band (band b2 to band b10) for control subjects (CS), patients with primary progressive MS (PPMS), and patients with relapsing-remitting MS (RRMS). Error bars represent standard errors

To investigate the association between altered ChP volume and tissue damage surrounding the ventricular system, we quantified microstructural tissue damage from the T1w/FLAIR ratios within periventricular bands of NAWM. As a first comparison of tissue damage between the CS, RRMS, and PPMS groups, we averaged T1w/FLAIR ratios within each band and observed an overall reduced mean ratio in RRMS patients when compared to the CS group and even smaller T1w/FLAIR ratios for PPMS patients (Fig. 4). Furthermore, with further distances from the ventricular margin, the T1W/FLAIR ratios in the patient groups approximated to those seen in the CS group. In comparison, the CS group exhibited a decreasing average T1w/FLAIR ratio in the first periventricular bands b2 to b5 but reached stability in the last five bands. We also observed an almost stable state for RRMS in bands b4 to b10; however, this was not the case for PPMS patients, who showed a continuous periventricular gradient towards the levels seen in patients with RRMS. Underlying pairwise comparisons between each band and each group is summarized in the supplement (Supplement Fig. 5, Supplement Table 1) with Supplement Fig. 5 showing the results from a linear mixed model analysis that included age, sex, and center as fixed effects and subjects as random effect.

### The relationship between T1w/FLAIR ratios and choroid plexus volume

**Fig. 5:**
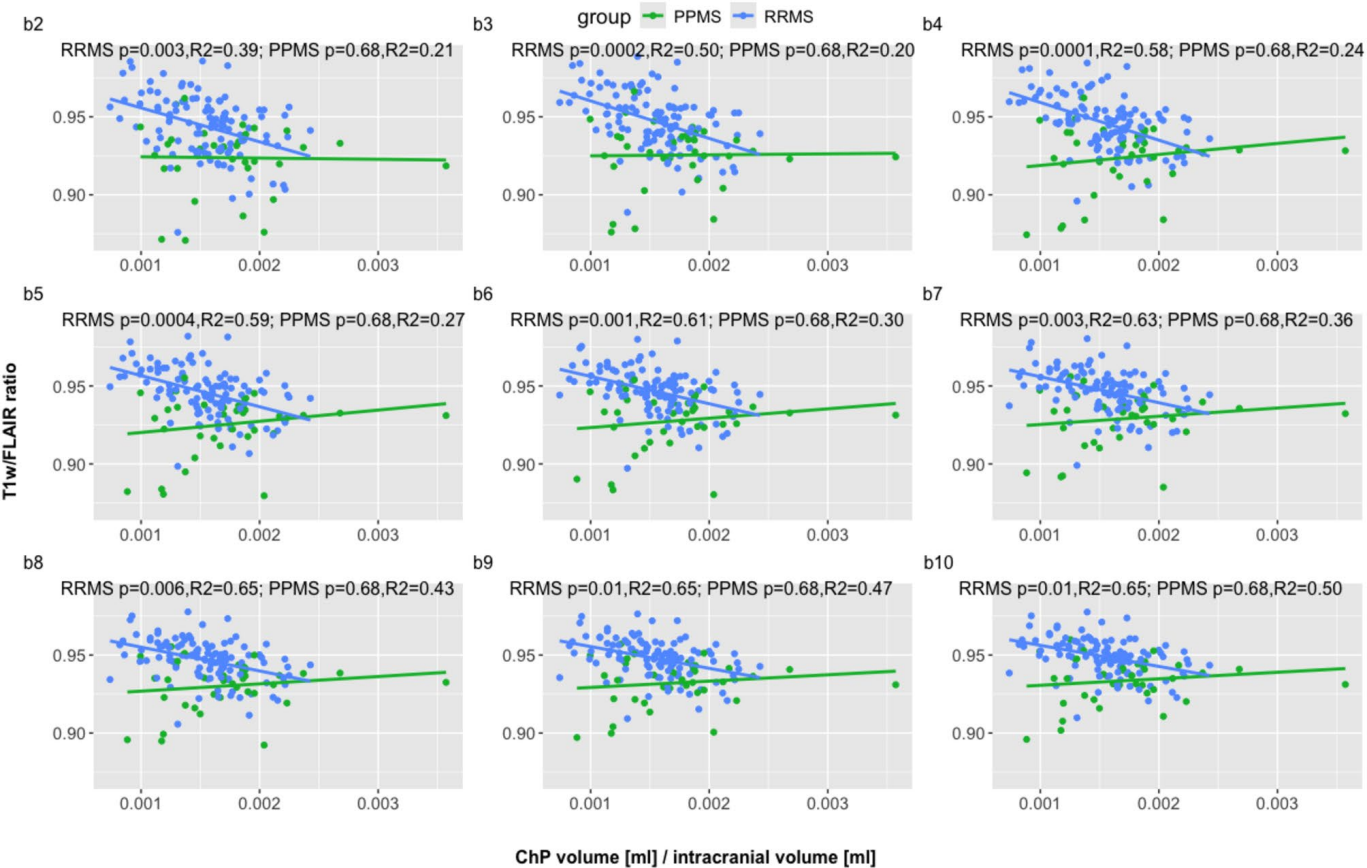
**T1w/FLAIR ratios versus ChP volumes for each periventricular band**. Relationship between T1w/FLAIR ratios and ChP volumes normalized to intracranial volume in each periventricular band (band b2 to band b10) for relapsing-remitting MS (RRMS,blue) and primary progressive MS (PPMS, green) patients. *P*-values (for ratios vs. ChP volumes) and adjusted R2 were obtained from linear regression analyses with age, sex, and center as covariates. P-values were corrected for multiple comparisons using Benjamini-Hochberg procedure

Next, we investigated the relationship between ChP and tissue damage, as reflected by the T1w/FLAIR ratios, for the patient groups (Fig. 5). ChP enlargement was significantly associated with decreasing T1w/FLAIR ratios in all periventricular bands in RRMS, but not in PPMS. With increasing distance from the ventricles, the relationship in RRMS was flattened as the slope continuously decreased after band four from − 14.6 in band b4 to -5.8 in band b10.

In both patient groups, T1w/FLAIR ratios in the whole WM were significantly associated with lesion volume, WM volume, and cortical GM volume (Suppl. Figure 6).

### T1w/FLAIR ratios and disease duration

**Fig. 6:**
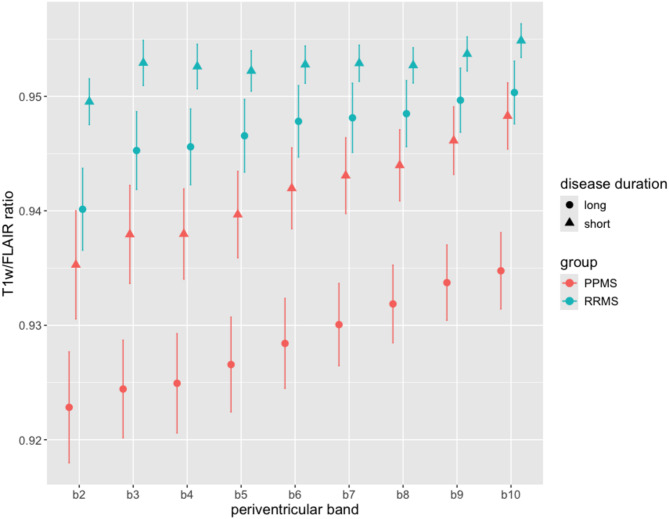
**T1w/FLAIR ratios for long and short disease durations**. Mean T1w/FLAIR ratios in each periventricular band (band b2 to band b10) for relapsing-remitting MS (RRMS) and primary progressive MS (PPMS) patients divided into long (>10 years) and short (<10 years) disease durations. Error bars represent standard errors

To further identify disease-specific differences in tissue damage between RRMS and PPMS patients, we analysed the T1w/FLAIR ratios in relation to disease duration. First, we divided each patient group into patients with a short and long disease duration with a cut-off of 10 years. Stratifying the patient groups with a disease duration threshold that was less than 10 years was not appropriate for our study cohort due to inconsistent sample sizes. The final division resulted in groups of 80 RRMS and 21 PPMS patients with a short disease duration (RRMS_sd, PPMS_sd) and 36 RRMS and 21 PPMS patients with a long disease duration (RRMS_ld, PPMS_ld). Further analysis showed that RRMS and PPMS patients with a short disease duration had higher T1w/FLAIR ratios compared to those with a long disease duration (Fig. 6). With increasing distance from the ventricles, PPMS_sd reached the level of RRMS_ld. In addition, with increasing distance from the ventricles, the PPMS group showed a stable condition with regards to the difference in T1w/FLAIR ratios between the two disease duration groups. In contrast, the differences between short and long disease duration in the RRMS group was larger in the inner periventricular bands (b2 to b5) than in more distant bands (Fig. 6).

## Discussion

In this study, we investigated the role of the ChP in neuroinflammatory activity in MS in both the relapsing-remitting and the primary progressive type of MS.

In a recent study, Fleischer et al. [6] showed that ChP volumes, as assessed by MRI volumetric analyses, were increased in patients with relapsing-remitting MS (RRMS) and were correlated with both disease activity and clinical disability. These authors proposed that ChP volumetry represents a promising marker to assess treatment response in MS, especially for those approaches that directly interact at the BCSFB [6]. Previous research suggested that the nature of inflammation and the targets of pathological processes might exhibit alterations during disease and in different types of MS [18]. Thus, during the relapsing-remitting phase of the disease, which manifests as the inflammation-driven subtype of MS, treatment options that modulate immune responses are available. However, these therapies are less effective for progressive forms of MS as the inflammation might be inaccessible to immune treatments in these particular stages [19]. Over recent years, it has been widely accepted that, similar to RRMS, the progressive type of MS also has an inflammatory component, although the inflammatory reactions that occur in this subtype appears to be compartmentalized from damage to the blood-brain barrier (BBB) [18, 20]. To further investigate this inflammatory component, it is of crucial importance to investigate the relevance of ChP as a biomarker for inflammatory processes in the progressive phase of MS to evaluate treatment options.

First, we confirmed ChP enlargement in a large sample of RRMS patients concurring previous studies [6, 8]. This highlighted the relevance of volumetric alterations of the ChP as quantified by MRI as a promising biomarker for disease activity in RRMS. Second, although the average ChP volume was similar to the RRMS group, we did not provide evidence for significant ChP enlargement in PPMS patients. This finding may be related to less statistical power due to the smaller sample size in the PPMS group, but may also indicate differing inflammatory components in the two disease subtypes, underlining the view of PPMS being a primary neurodegenerative disease with less inflammatory focus. Although our findings did not identify the ChP as a biomarker for treatment in PPMS, it may point to major differences between the two disease types regarding their neuroinflammatory behaviours.

In general, the chronic phase of the disease is thought to have a minor inflammatory component. Both the BBB and the BCSFB undergo alterations under neuroinflammatory conditions: A BBB breakdown and vascular leackage is induced by altered junctional molecules and leukocyte recruitment is driven by endothelial activation [21]. For the BCSFB, which includes both the pial vessels in the leptomeningeal compartment and the epithelial cells in the choroid plexus, a loss of an essential tight junction protein has been described in MS leading to an enhanced leukocyte infiltration into the CSF and brain parenchyma [22, 23]. Pathological processes in chronic MS has been described to be less associated with BBB alterations than in RRMS [18, 19] and similar differences might be observed for BCSFB. According to Lassmann et al. [18], there are two different pathophysiological inflammatory pathways that each occur to differing degrees in different subtypes of MS. The first (peripheral) mechanism that dominates in RRMS is associated with a major disturbance of the BBB, the involvement of ChP inflammation, and the formation of new focal lesions. The second (central) mechanism is defined by a diffuse inflammatory reaction that occurs predominantly in the meninges and perivascular spaces and is associated with slowly expanding lesions in the WM; this mechanism is typically involved in progressive MS. Nevertheless, no sharp distinction can be made between the relative involvements of these two pathological pathways in the subtypes of MS. Notably, both pathophysiological pathways appear to exert impact in early PPMS. Our group comparisons support the hypothesis that the peripheral mechanism may relate to ChP enlargement in RRMS while the central mechanism predominates in PPMS.

However, age-related neurodegenerative effects should be considered when interpreting potential differences between the disease types. In our study, PPMS patients were older than CS and RRMS patients, which could influence ChP volume changes. Age was included as a confounding factor in our analysis, which substantially changed the results as differences between PPMS and CS were significant in the sub-analysis without adding age as a covariate. On the one hand, this supported the previously shown association between ChP volume and age-related neurodegeneration, which showed a non-linear increase of ChP volume with age [24, 25]. The increase of ChP volume seemed to start at an age of 55 years, which was not present or milder in younger subjects. Since our CS and RRMS patients were younger than this threshold of 55 years on average, this age-related effect might not affect our study sample and the differences between RRMS and CS. On the other hand, this strengthened the relationship to disease-specific neuroinflammatory processes in RRMS, where significant differences were found independent of ageing. Thus, although ChP volumes might be affected by ageing, it seemed to also have a component driven by other pathophysiological mechanisms.

For the interpretation of enlarged ChP volumes vascular changes might also be taken into account, which was investigated in recent studies [24, 26]. The dysfunction of molecules, including arginine vasopressin, dopamine, serotonin, with increasing age is responsible for reduced CBF in the ChP and oxygen and nutrient delivery as well as metabolic clearance may lead to damage and neurodegeneration. Furthermore, Eisma et al. (2023) found that smaller ChP volumes were associated with increased perfusion and enlarged ChP volumes corresponded to decreased perfusion. Similar to aging, neuroinflammatory processes and associated enhanced leukocyte infiltration into the CSF might require an increased blood flow which would be in turn associated with enlarged ChP volumes.

Our analyses also identified a relationship between disease duration and increasing ChP volumes in patients with RRMS but not those with PPMS. Although we did not study longitudinal MRI data, we interpret these findings as an indication that inflammatory processes in RRMS patients first increase before reaching a stable status, in which the ChP volumes seemed to be similar to the progressive patients’ ChP volumes. This suggested that plexus enlargement and inflammation follow a dynamic process. However, the acquisition of longitudinal data would be necessary to confirm ChP involvement over time for both disease types and to disentangle the relevance of ChP volume changes with regards to the conversion of RRMS patients to SPMS.

We identified an association between LVV and ChP for RRMS, but not PPMS, which might be driven by LVV values smaller than 20 ml. This might be one explanation why RRMS and not PPMS showed this relationship because smaller ventricles were mainly present in RRMS. On the one hand, larger ventricles might need larger plexus volumes because of increased CSF production. On the other hand, there might also be a physiological association which means that the choroid plexus might be stretched along the larger ventricle. However, larger ventricular volume represents increased atrophy, which is therefore connected to white matter and grey matter volumes. Thus, no sharp distinction can be made between these parameters.

Furthermore, we identified an association between ChP volumes and lesion load in RRMS patients showing increased lesion volume with enlarging ChP volumes. As lesion load represents a marker for disease activity and inflammation, this relationship, which has already been shown by previous studies [27–29] highlighted the role of ChP in inflammatory processes. Especially iron rim lesions which reflect a chronic active inflammation have been shown to be associated with ChP volume [27, 30]. Since both markers have microglia activity in common, similar pathological mechanisms might be responsible in both chronic lesion inflammation and ChP enlargement [27]. In our study, no significant association between WM and ChP volumes were found, which might serve as an indirect confirmation that those brain areas, which are less affected by inflammatory processes than lesions and which also have less borders to the CSF compartment, are less affected by neuroinflammation from the ChP. Another plausible association to other brain structures is the negative relationship between ChP and cortex volume in our RRMS group. This suggested that due to its proximity to cerebrospinal fluid, the cortex might represent a vulnerable structure that is more affected with higher inflammation represented by enlarged ChP volumes. Not only periventricular but also cortical gradients of brain parenchymal damage have been shown to exist in MS, which were summarized as surface-in gradients and might reflect similar processes based on their proximity to the CSF compartment [31]. Still, the relationship between CSF-derived factors and tissue pathology remains uncertain, both at the border between CSF and the ventricular ependyma and between CSF and the meninges whose inflammation is seen in all types of MS [32].

In a recent positron-emission tomography study, microglial activation was found to exhibit a periventricular gradient suggesting a link to the proximity to the ventricular CSF [33]. Identifying the potential role of pro-inflammatory factors from the CSF is of crucial importance. The ChP is the key structure responsible for the production of CSF and of a variety of immune cells that were recently shown to be associated with reduced levels of periventricular remyelination [34]. Although those immune cells enter the brain through meningeal blood vessels [1], the proximity to CSF might be a possible link between periventricular and cortical gradients [31]. Thus, there might also be an association between increased inflammatory mechanisms, as quantified by ChP volumes, on those microstructures of the brain that are closely associated with the ventricular system. In our RRMS patient group, ChP enlargement was found to be related to demyelination in the NAWM, as represented by T1w/FLAIR ratios of the periventricular bands. This relationship was getting weaker with increasing distance to the ventricles, which suggested that ChP inflammation has greater significant impact on demyelination or tissue damage in periventricular NAWM regions. Pro-inflammatory environments evolving from the ChP could induce a reduction of remyelination, as suggested previously [34]. In PPMS, periventricular NAWM abnormalities might be rather associated with cortical pathology [35], which is more prominent during progressive MS [36], than with ChP enlargements. Further analysis might be necessary to elucidate the relationship between cortical pathology and ChP volumes, especially in PPMS.

The interpretation of T1w/FLAIR or magnetization transfer ratio (MTR) as a means of representing tissue damage has been debated at length, particularly in terms of whether this methodology can reflect myelin content or rather axonal damage and microstructural tissue alterations [9]. Previously, image ratios of T1w/T2w contrast were used to evaluate tissue damage and demyelination as these ratios were considered to reflect increased myelin contrast [37, 38]. However, current MRI guidelines for MS suggest that three-dimensional (3D) FLAIR sequences should be utilized in the standard protocol since lesions are preferably detected on FLAIR images [39]. Therefore, the quantification of T1w/FLAIR images from existing FLAIR data could be used to quantify tissue damage without requiring additional quantitative sequences. It has previously been shown that within periventricular NAWM, a gradient exists showing pathology that decreases with longer distance from the ventricles [40–42]. In our study, we observed reduced T1w/FLAIR ratios in NAWM in both MS patient groups when compared with controls. Furthermore, as a proof of concept, we were able to show that T1w/FLAIR in NAWM was highly correlated with normalized brain WM and GM volumes and with the total lesion volume, as would be expected for a measure of myelin tissue content.

In our analyses of periventricular gradients of tissue involvement PPMS showed the lowest T1w/FLAIR values in each periventricular band. This finding was consistent with the conclusions published previously by Liu et al. [40] who showed that the MTR in NAWM represented myelin content and was lower in MS than in controls; furthermore, in that study, the periventricular reduction was greater in SPMS than in RRMS.

Moreover, we confirmed that a greater disease related effect seemed to occur on the inner surface of the brain close to the ventricles as the average T1w/FLAIR ratios gradually increased in more distant periventricular bands. A corresponding effect has already been described in previous studies, in which a periventricular gradient of microstructural tissue damage was quantified by MTR [31, 33, 40]. In the present study, both the control and RRMS groups reached a stable condition of T1w/FLAIR ratios at longer distances from the ventricles, whereas PPMS patients showed a continuous increase. This finding suggested that in the PPMS group, but not the RRMS and control groups, tissue changes in NAWM may extend to areas that are more distant from the ventricular margin and that a constant level was not attained in PPMS within the pre-set 10 mm distance. In CS, the decrease of T1/FLAIR ratios close to the ventricles was in line with previous studies showing MTR values in NAWM of healthy controls also declining with distance from the ventricles [40]. This might highlight that under healthy conditions, periventricular areas, which include for example the corpus callosum, are highly myelinated and that this declined in deeper WM structures. In contrast, highly myelinated periventricular areas seemed to suffer most from damage in patients.

Interestingly, separating patient groups into short and long disease duration showed that the T1w/FLAIR ratios of PPMS patients with short disease duration approached the levels of RRMS patients with long disease duration at longer distances from the ventricles. This suggests that the periventricular gradient of tissue damage in NAWM becomes more pronounced as the disease progresses, and that the outer regions are affected to a similar extent in early-onset PPMS patients as in long-standing RRMS patients.

The consistency of our results with the cited previous studies regarding the detection of a periventricular gradient of tissue impairment in MS and our observation of disease-specific reductions of T1w/FLAIR ratios in NAWM highlighted the suitability of the T1w/FLAIR ratio as a marker for tissue abnormalities and its comparability with MTR measures. Similarly, a recent longitudinal study by Tonietto et al. [34], involving patients with MS, supported the concept of a periventricular gradient. They showed that greater demyelination was observed in MS lesions close to the ventricles and that the probability for remyelination was higher with increasing distance from the ventricles. Previous studies suggested that the periventricular areas are characterized by a high venous density [43] and concurrent low oxygenation, thus leading to a high density of WM lesions with a simultaneous reduced ability to remyelinate [34]. This is in line with a histopathological study showing less remyelination in periventricular plaques compared to subcortical or deep white matter [44]. Furthermore, the diffusion of pro-inflammatory factors derived from the CSF into surrounding regions may be able to influence the course of MS lesion development, as these factors could induce a pro-inflammatory environment that impedes remyelination [45].

In our analysis, we failed to identify any relationship between ChP and the severity of clinical disability, as quantified by EDSS. It is possible that the current clinical scales may not be specific enough for a structure as small as the ChP, which could represent a marker for disease activity, but not for the overall degree of disability. However, this association was demonstrated successfully in a previous study [6]. One reason for this discrepancy might be the differential selection of MS patients. Fleischer et al. [6] only focused on clinically isolated syndrome (CIS) and RRMS patients whereas our dataset al.so included patients with chronic MS. Another reason for the lack of association between the ChP, chronic inflammation, and clinical disability, might be the impact of spinal cord pathology on EDSS which predominates in progressive MS stages.

When interpreting our results, some limitations must be considered. First, the classification of our patients into short and long disease durations was based on a relatively long period of 10 years. Future studies should compare more differentiated groups of disease duration, such as ≤ 5 years, 5 to 10 years, or > 10 years. Second, longitudinal data should provide the basis for further analyses of the chronological order in which inflammation and microstructural damage in MS occur and affect each other. Third, the T1w/FLAIR results in the second periventricular band may be influenced by partial volume effects, at least in part, and should be interpreted with caution. Fourth, we did not perform a direct comparison of our T1w/FLAIR results with established myelin measures, as for example T1w/T2w ratios, myelin maps from quantitative MRI, MTR, or metrics from diffusion tensor imaging (DTI). Instead, we referred to the methodology described by Cappelle et al. [9] who demonstrated a good correlation between T1w/FLAIR and MTR as a measure of myelin content and microstructural tissue integrity. Since it is, to our knowledge, the only study describing T1w/FLAIR ratios as a sensitive biomarker in WM disorders, future studies should include an independent evaluation of the T1w/FLAIR ratio with other myelin measures, or post-mortem data. So far, no correlations to other myelin measures have been described.

## Conclusions

This study showed that enlargement of the ChP is associated with periventricular tissue damage in RRMS, which highlighted the relevance of neuroinflammation emerging from the CSF for demyelinating processes. Moreover, our analyses provided evidence for distinct processes occurring in relapsing-remitting and primary-progressive MS with regard to neuroinflammation over time. ChP enlargement was increased in RRMS, but not in PPMS patients, when compared to healthy controls. This highlights the predominate neurodegenerative component of the progressive disease phase. We further observed the importance of the ChP in relapsing-remitting MS during inflammatory processes, which showed not only a relationship to lesion load, but also to cortical structures that might be affected concurrently by meningeal inflammation.

## Electronic supplementary material

Below is the link to the electronic supplementary material.

Supplementary Material 1

## Data Availability

The datasets for this article are not publicly available due to concerns regarding participant anonymity.

## Abbreviations

ANCOVA: Analysis of covariance
BBB: Blood-brain barrier
BCSFB: Blood-cerebrospinal fluid barrier
ChP: Choroid plexus
CS: Control subjects
DMT: Disease-modifying therapies
EDSS: Expanded disability status scale
FLAIR: Fluid-attenuated inversion recovery
LV: Lateral ventricles
MTR: Magnetization transfer ratio
NAWM: Normal appearing white matter
PPMS: Primary progressive multiple sclerosis
RRMS: Relapsing-remitting multiple sclerosis
T1w: T1-weighted
