# Supplementary material for: Relevance of choroid plexus volumes in multiple sclerosis
Source: Fluids Barriers CNS. 2025 May 8;22:47. doi: 10.1186/s12987-025-00656-7 (PMC12060557; doi:10.1186/s12987-025-00656-7)
Supplement: Supplementary file 1 — Supplementary Material 1 [file 12987_2025_656_MOESM1_ESM.docx]

# Supplement for manuscript “Relevance of choroid plexus volumes in multiple sclerosis” (B. Krieger et al.)


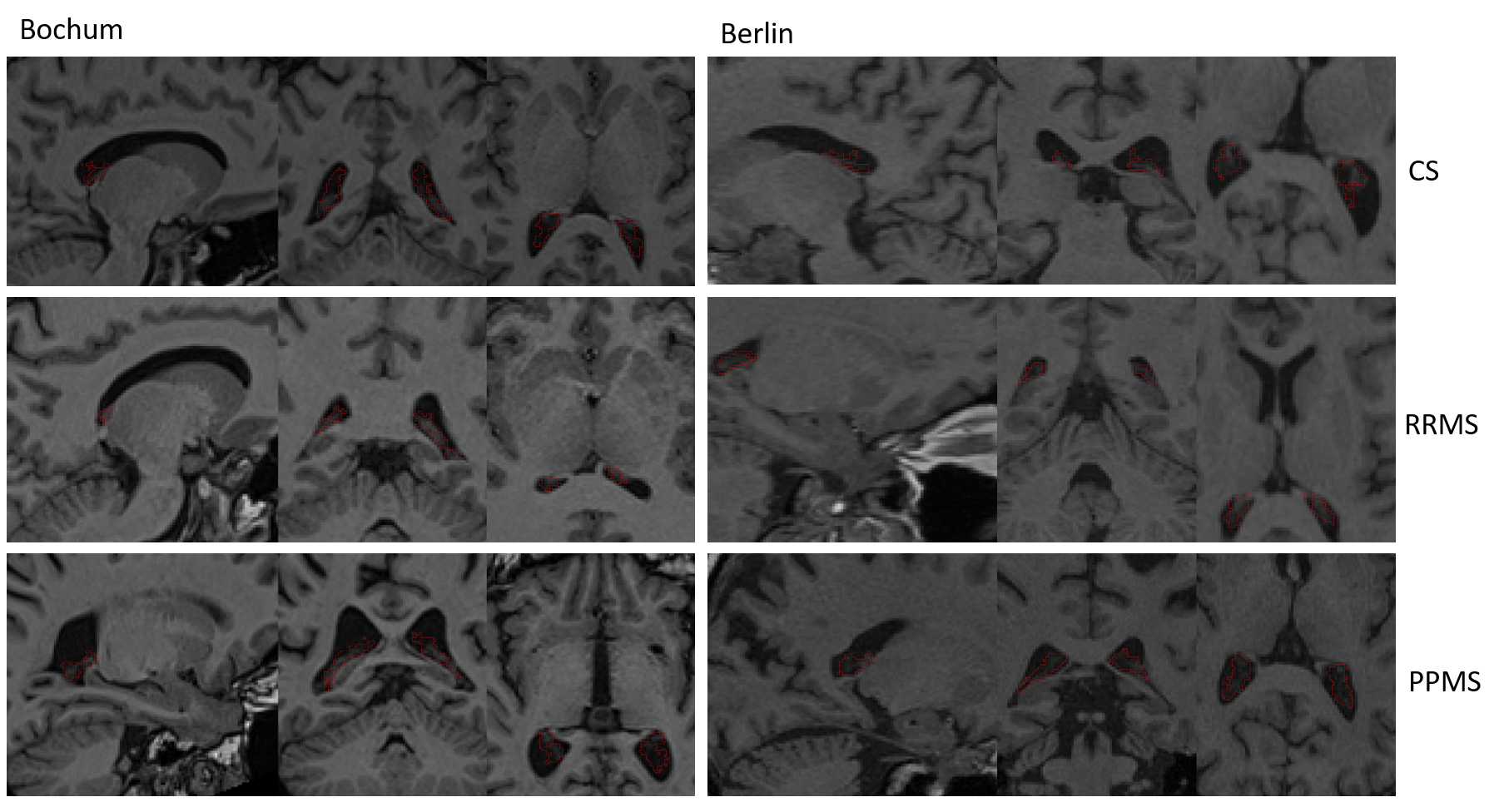


**Supplement Figure 1** Examples of choroid plexus segmentations (red outline) for both centres (left: Bochum, right: Berlin) and each subject group (first row: CS, second row: RRMS, third row: PPMS).


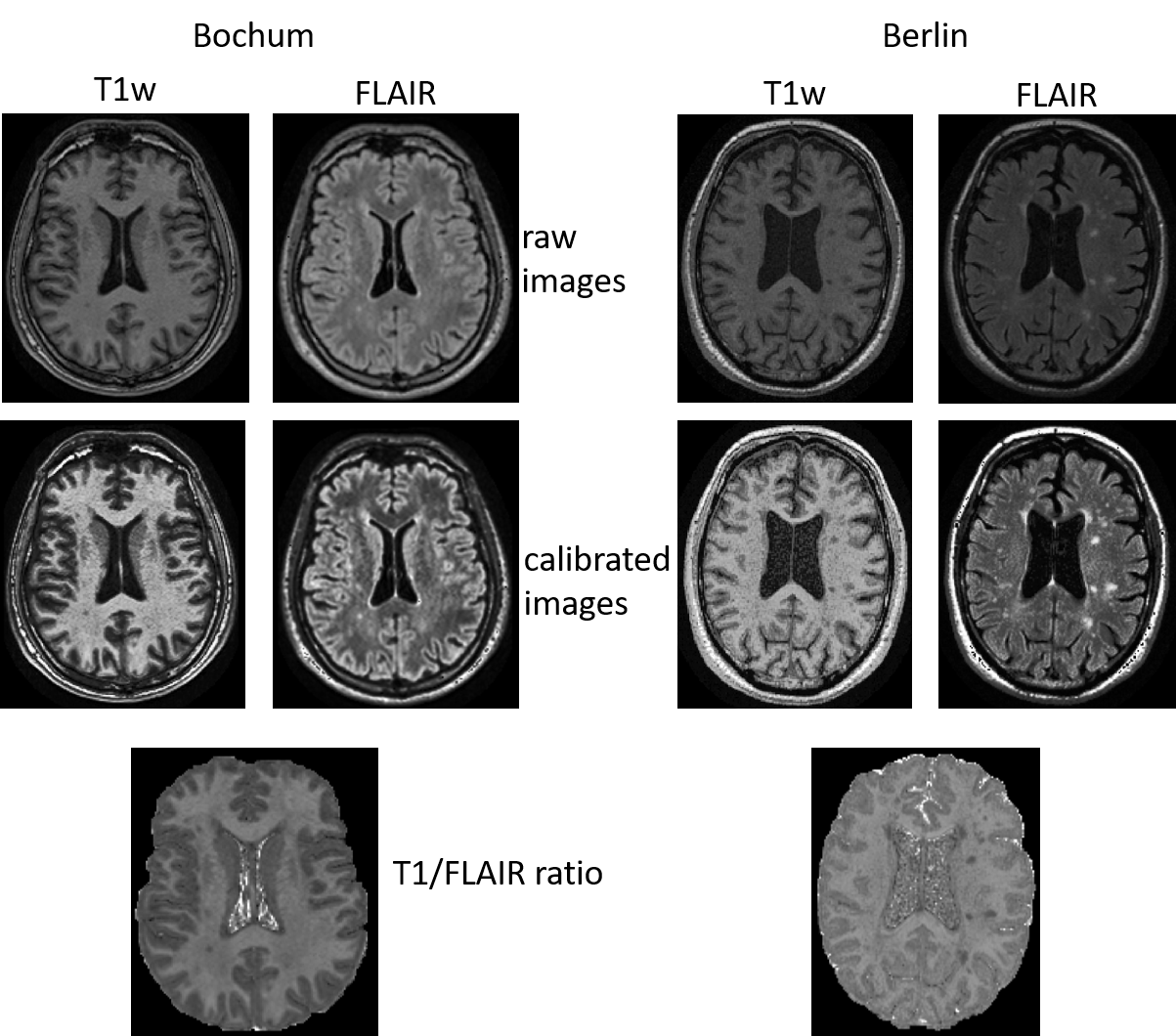


**Supplement Figure 2** Examples of raw T1w-weighted and FLAIR images, calibrated images, and T1/FLAIR ratio images for both centers (left: Bochum, right: Berlin).


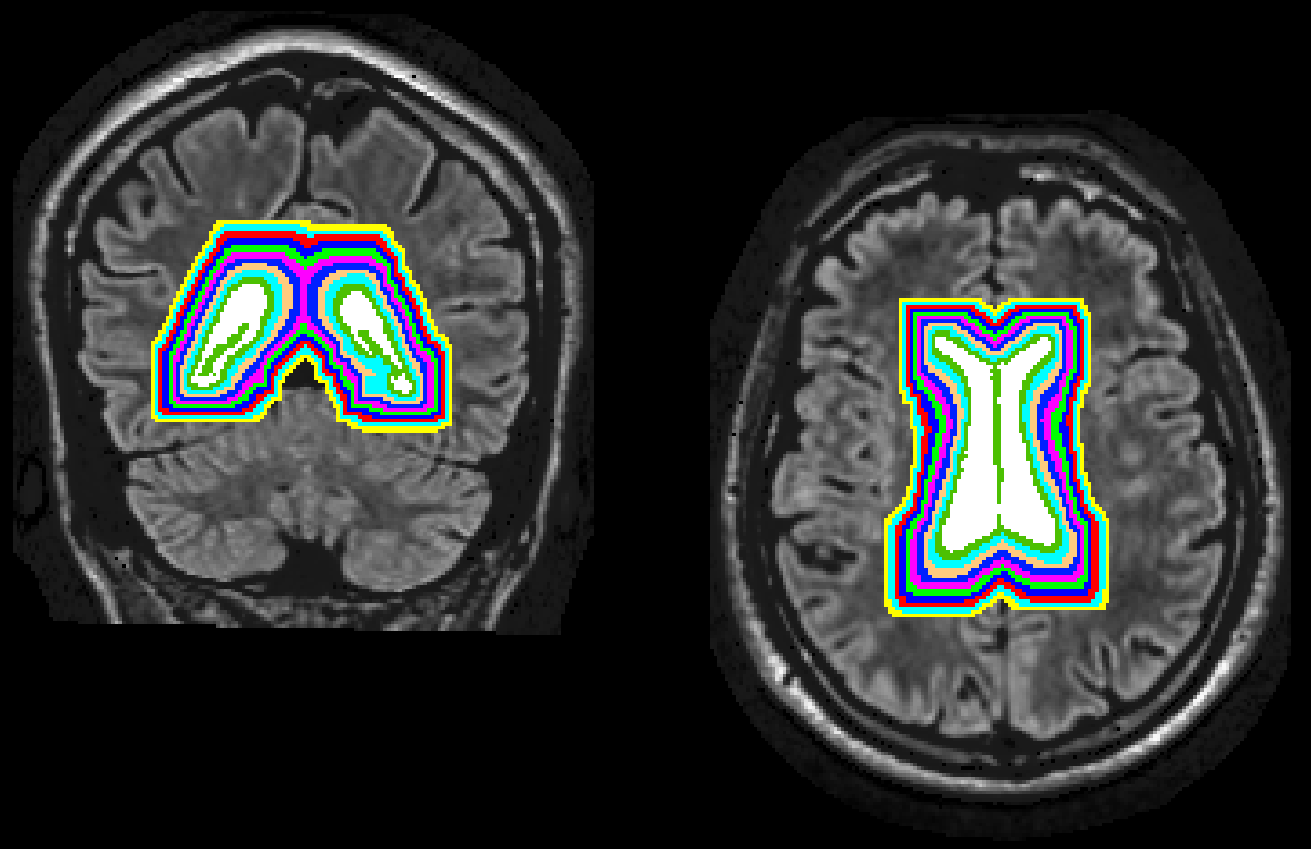


**Supplement Figure 3** **Periventricular bands.** Ten periventricular bands in NAWM were created by was repeatedly dilating the supratentorial ventricle mask by one-voxel (1 x 1 x 1 mm3). The band masks were further multiplied by the brain WM mask to exclude lesions and the cortex.


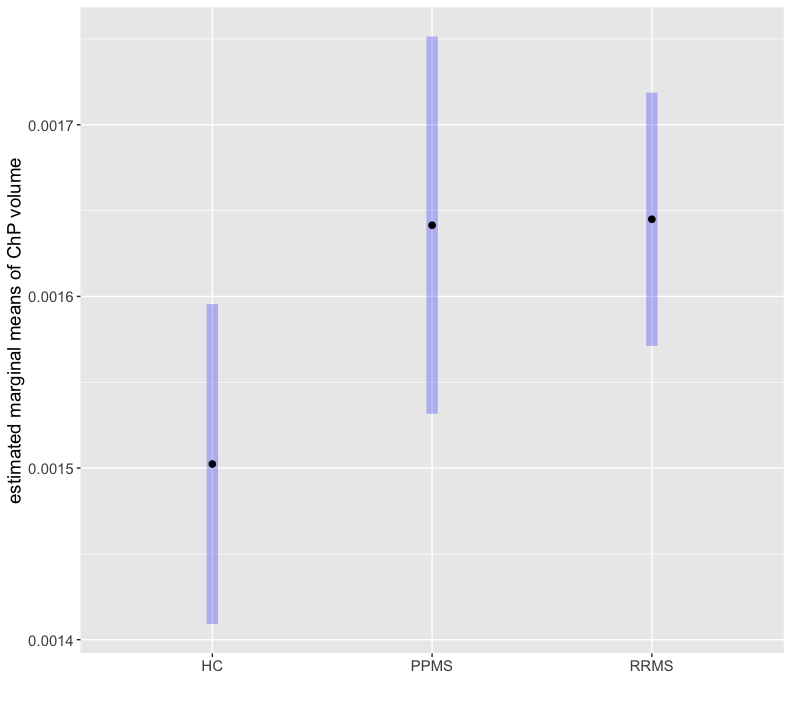


**Supplement Figure 4** Comparison of choroid plexus (ChP) volumes between control subjects (CS), primary progressive MS (PPMS), and relapsing-remitting MS (RRMS). Values represent estimated marginal means from pairwise post-hoc tests after analysis of covariance analysis with age, sex, and center as covariates.


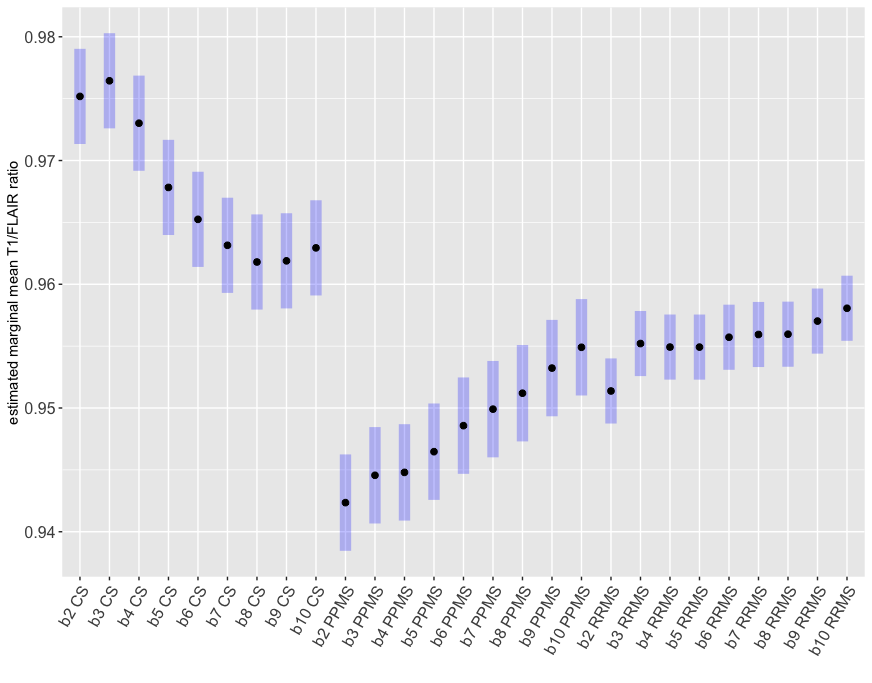


**Supplement Figure 5** Visualization of estimated marginal means from mixel model analysis with age, sex, and center as fixed effects, and subject as random effects for comparisons of T1/FLAIR ratios between each periventricular band (b2 to b10) and each group (control subjects (CS), primary progressive MS (PPMS), relapsing-remitting MS (RRMS)). Corresponding p-values and estimated differences for significant differences are summarized in Supplement Table 1.

**
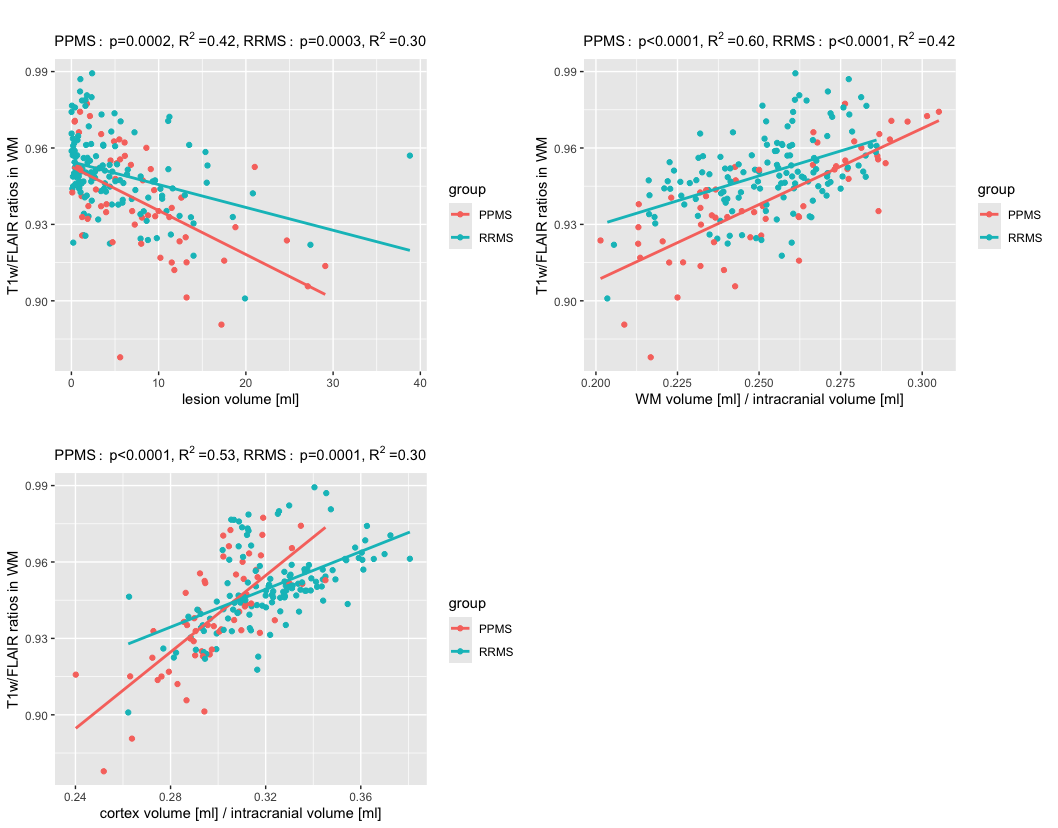
**

**Supplement Figure 6 T1w/FLAIR ratios in normal appearing white matter (NAWM) versus lesion volume, WM volume, and cortical grey matter (GM) volume.** Relationship between the mean T1w/FLAIR ratios in the whole NAWM and lesion, normalised WM, or GM volume for primary progressive (PPMS) and relapsing-remitting (RRMS) patients. P-values (for ratios vs. each volume) and adjusted R2 were obtained from linear regression analyses with age and sex as covariates.

**Supplement Table 1** Significant results from mixel model analysis with age, sex, and center as fixed effects, and subject as random effects for comparisons of T1/FLAIR ratios between each band (b2 to b10) and each group (control subjects (CS), primary progressive MS (PPMS), relapsing-remitting MS (RRMS)) (p < 0.05). P values of 0.000 represent values smaller than 0.001.

| contrast | estimate | SE | p.value |
| --- | --- | --- | --- |
| b2 CS - b5 CS | 0.00735633 | 0.00111283 | 0.000 |
| b2 CS - b6 CS | 0.00993416 | 0.00111283 | 0.000 |
| b2 CS - b7 CS | 0.01203236 | 0.00111283 | 0.000 |
| b2 CS - b8 CS | 0.01338131 | 0.00111283 | 0.000 |
| b2 CS - b9 CS | 0.01328809 | 0.00111283 | 0.000 |
| b2 CS - b10 CS | 0.01223973 | 0.00111283 | 0.000 |
| b2 CS - b2 PPMS | 0.03282726 | 0.00291612 | 0.000 |
| b2 CS - b3 PPMS | 0.03061846 | 0.00291612 | 0.000 |
| b2 CS - b4 PPMS | 0.03038355 | 0.00291612 | 0.000 |
| b2 CS - b5 PPMS | 0.02871075 | 0.00291612 | 0.000 |
| b2 CS - b6 PPMS | 0.02660619 | 0.00291612 | 0.000 |
| b2 CS - b7 PPMS | 0.02527338 | 0.00291612 | 0.000 |
| b2 CS - b8 PPMS | 0.02398545 | 0.00291612 | 0.000 |
| b2 CS - b9 PPMS | 0.02195533 | 0.00291612 | 0.000 |
| b2 CS - b10 PPMS | 0.02027453 | 0.00291612 | 0.000 |
| b2 CS - b2 RRMS | 0.0238051 | 0.00214992 | 0.000 |
| b2 CS - b3 RRMS | 0.0199717 | 0.00214992 | 0.000 |
| b2 CS - b4 RRMS | 0.02025653 | 0.00214992 | 0.000 |
| b2 CS - b5 RRMS | 0.02025987 | 0.00214992 | 0.000 |
| b2 CS - b6 RRMS | 0.01946164 | 0.00214992 | 0.000 |
| b2 CS - b7 RRMS | 0.01924014 | 0.00214992 | 0.000 |
| b2 CS - b8 RRMS | 0.01921612 | 0.00214992 | 0.000 |
| b2 CS - b9 RRMS | 0.01815671 | 0.00214992 | 0.000 |
| b2 CS - b10 RRMS | 0.01712017 | 0.00214992 | 0.000 |
| b3 CS - b5 CS | 0.00861911 | 0.00111283 | 0.000 |
| b3 CS - b6 CS | 0.01119695 | 0.00111283 | 0.000 |
| b3 CS - b7 CS | 0.01329515 | 0.00111283 | 0.000 |
| b3 CS - b8 CS | 0.01464409 | 0.00111283 | 0.000 |
| b3 CS - b9 CS | 0.01455087 | 0.00111283 | 0.000 |
| b3 CS - b10 CS | 0.01350251 | 0.00111283 | 0.000 |
| b3 CS - b2 PPMS | 0.03409004 | 0.00291612 | 0.000 |
| b3 CS - b3 PPMS | 0.03188125 | 0.00291612 | 0.000 |
| b3 CS - b4 PPMS | 0.03164633 | 0.00291612 | 0.000 |
| b3 CS - b5 PPMS | 0.02997353 | 0.00291612 | 0.000 |
| b3 CS - b6 PPMS | 0.02786897 | 0.00291612 | 0.000 |
| b3 CS - b7 PPMS | 0.02653616 | 0.00291612 | 0.000 |
| b3 CS - b8 PPMS | 0.02524823 | 0.00291612 | 0.000 |
| b3 CS - b9 PPMS | 0.02321811 | 0.00291612 | 0.000 |
| b3 CS - b10 PPMS | 0.02153731 | 0.00291612 | 0.000 |
| b3 CS - b2 RRMS | 0.02506788 | 0.00214992 | 0.000 |
| b3 CS - b3 RRMS | 0.02123448 | 0.00214992 | 0.000 |
| b3 CS - b4 RRMS | 0.02151932 | 0.00214992 | 0.000 |
| b3 CS - b5 RRMS | 0.02152265 | 0.00214992 | 0.000 |
| b3 CS - b6 RRMS | 0.02072442 | 0.00214992 | 0.000 |
| b3 CS - b7 RRMS | 0.02050292 | 0.00214992 | 0.000 |
| b3 CS - b8 RRMS | 0.0204789 | 0.00214992 | 0.000 |
| b3 CS - b9 RRMS | 0.01941949 | 0.00214992 | 0.000 |
| b3 CS - b10 RRMS | 0.01838295 | 0.00214992 | 0.000 |
| b4 CS - b5 CS | 0.00519167 | 0.00111283 | 0.001 |
| b4 CS - b6 CS | 0.00776951 | 0.00111283 | 0.000 |
| b4 CS - b7 CS | 0.00986771 | 0.00111283 | 0.000 |
| b4 CS - b8 CS | 0.01121665 | 0.00111283 | 0.000 |
| b4 CS - b9 CS | 0.01112344 | 0.00111283 | 0.000 |
| b4 CS - b10 CS | 0.01007507 | 0.00111283 | 0.000 |
| b4 CS - b2 PPMS | 0.03066261 | 0.00291612 | 0.000 |
| b4 CS - b3 PPMS | 0.02845381 | 0.00291612 | 0.000 |
| b4 CS - b4 PPMS | 0.02821889 | 0.00291612 | 0.000 |
| b4 CS - b5 PPMS | 0.0265461 | 0.00291612 | 0.000 |
| b4 CS - b6 PPMS | 0.02444154 | 0.00291612 | 0.000 |
| b4 CS - b7 PPMS | 0.02310872 | 0.00291612 | 0.000 |
| b4 CS - b8 PPMS | 0.02182079 | 0.00291612 | 0.000 |
| b4 CS - b9 PPMS | 0.01979067 | 0.00291612 | 0.000 |
| b4 CS - b10 PPMS | 0.01810988 | 0.00291612 | 0.000 |
| b4 CS - b2 RRMS | 0.02164044 | 0.00214992 | 0.000 |
| b4 CS - b3 RRMS | 0.01780704 | 0.00214992 | 0.000 |
| b4 CS - b4 RRMS | 0.01809188 | 0.00214992 | 0.000 |
| b4 CS - b5 RRMS | 0.01809522 | 0.00214992 | 0.000 |
| b4 CS - b6 RRMS | 0.01729698 | 0.00214992 | 0.000 |
| b4 CS - b7 RRMS | 0.01707549 | 0.00214992 | 0.000 |
| b4 CS - b8 RRMS | 0.01705147 | 0.00214992 | 0.000 |
| b4 CS - b9 RRMS | 0.01599205 | 0.00214992 | 0.000 |
| b4 CS - b10 RRMS | 0.01495551 | 0.00214992 | 0.000 |
| b5 CS - b7 CS | 0.00467604 | 0.00111283 | 0.008 |
| b5 CS - b8 CS | 0.00602498 | 0.00111283 | 0.000 |
| b5 CS - b9 CS | 0.00593176 | 0.00111283 | 0.000 |
| b5 CS - b10 CS | 0.0048834 | 0.00111283 | 0.004 |
| b5 CS - b2 PPMS | 0.02547093 | 0.00291612 | 0.000 |
| b5 CS - b3 PPMS | 0.02326214 | 0.00291612 | 0.000 |
| b5 CS - b4 PPMS | 0.02302722 | 0.00291612 | 0.000 |
| b5 CS - b5 PPMS | 0.02135442 | 0.00291612 | 0.000 |
| b5 CS - b6 PPMS | 0.01924986 | 0.00291612 | 0.000 |
| b5 CS - b7 PPMS | 0.01791705 | 0.00291612 | 0.000 |
| b5 CS - b8 PPMS | 0.01662912 | 0.00291612 | 0.000 |
| b5 CS - b9 PPMS | 0.014599 | 0.00291612 | 0.000 |
| b5 CS - b10 PPMS | 0.0129182 | 0.00291612 | 0.004 |
| b5 CS - b2 RRMS | 0.01644877 | 0.00214992 | 0.000 |
| b5 CS - b3 RRMS | 0.01261537 | 0.00214992 | 0.000 |
| b5 CS - b4 RRMS | 0.01290021 | 0.00214992 | 0.000 |
| b5 CS - b5 RRMS | 0.01290354 | 0.00214992 | 0.000 |
| b5 CS - b6 RRMS | 0.01210531 | 0.00214992 | 0.000 |
| b5 CS - b7 RRMS | 0.01188382 | 0.00214992 | 0.000 |
| b5 CS - b8 RRMS | 0.01185979 | 0.00214992 | 0.000 |
| b5 CS - b9 RRMS | 0.01080038 | 0.00214992 | 0.000 |
| b5 CS - b10 RRMS | 0.00976384 | 0.00214992 | 0.002 |
| b6 CS - b2 PPMS | 0.0228931 | 0.00291612 | 0.000 |
| b6 CS - b3 PPMS | 0.0206843 | 0.00291612 | 0.000 |
| b6 CS - b4 PPMS | 0.02044938 | 0.00291612 | 0.000 |
| b6 CS - b5 PPMS | 0.01877659 | 0.00291612 | 0.000 |
| b6 CS - b6 PPMS | 0.01667203 | 0.00291612 | 0.000 |
| b6 CS - b7 PPMS | 0.01533921 | 0.00291612 | 0.000 |
| b6 CS - b8 PPMS | 0.01405128 | 0.00291612 | 0.001 |
| b6 CS - b9 PPMS | 0.01202116 | 0.00291612 | 0.013 |
| b6 CS - b2 RRMS | 0.01387093 | 0.00214992 | 0.000 |
| b6 CS - b3 RRMS | 0.01003754 | 0.00214992 | 0.001 |
| b6 CS - b4 RRMS | 0.01032237 | 0.00214992 | 0.001 |
| b6 CS - b5 RRMS | 0.01032571 | 0.00214992 | 0.001 |
| b6 CS - b6 RRMS | 0.00952748 | 0.00214992 | 0.004 |
| b6 CS - b7 RRMS | 0.00930598 | 0.00214992 | 0.006 |
| b6 CS - b8 RRMS | 0.00928196 | 0.00214992 | 0.006 |
| b6 CS - b9 RRMS | 0.00822254 | 0.00214992 | 0.036 |
| b7 CS - b2 PPMS | 0.0207949 | 0.00291612 | 0.000 |
| b7 CS - b3 PPMS | 0.0185861 | 0.00291612 | 0.000 |
| b7 CS - b4 PPMS | 0.01835118 | 0.00291612 | 0.000 |
| b7 CS - b5 PPMS | 0.01667839 | 0.00291612 | 0.000 |
| b7 CS - b6 PPMS | 0.01457383 | 0.00291612 | 0.000 |
| b7 CS - b7 PPMS | 0.01324101 | 0.00291612 | 0.002 |
| b7 CS - b8 PPMS | 0.01195308 | 0.00291612 | 0.014 |
| b7 CS - b2 RRMS | 0.01177273 | 0.00214992 | 0.000 |
| b7 CS - b4 RRMS | 0.00822417 | 0.00214992 | 0.036 |
| b7 CS - b5 RRMS | 0.00822751 | 0.00214992 | 0.036 |
| b8 CS - b2 PPMS | 0.01944595 | 0.00291612 | 0.000 |
| b8 CS - b3 PPMS | 0.01723715 | 0.00291612 | 0.000 |
| b8 CS - b4 PPMS | 0.01700224 | 0.00291612 | 0.000 |
| b8 CS - b5 PPMS | 0.01532944 | 0.00291612 | 0.000 |
| b8 CS - b6 PPMS | 0.01322488 | 0.00291612 | 0.002 |
| b8 CS - b7 PPMS | 0.01189207 | 0.00291612 | 0.015 |
| b8 CS - b2 RRMS | 0.01042379 | 0.00214992 | 0.001 |
| b9 CS - b2 PPMS | 0.01953917 | 0.00291612 | 0.000 |
| b9 CS - b3 PPMS | 0.01733037 | 0.00291612 | 0.000 |
| b9 CS - b4 PPMS | 0.01709546 | 0.00291612 | 0.000 |
| b9 CS - b5 PPMS | 0.01542266 | 0.00291612 | 0.000 |
| b9 CS - b6 PPMS | 0.0133181 | 0.00291612 | 0.002 |
| b9 CS - b7 PPMS | 0.01198529 | 0.00291612 | 0.013 |
| b9 CS - b2 RRMS | 0.01051701 | 0.00214992 | 0.001 |
| b10 CS - b2 PPMS | 0.02058753 | 0.00291612 | 0.000 |
| b10 CS - b3 PPMS | 0.01837874 | 0.00291612 | 0.000 |
| b10 CS - b4 PPMS | 0.01814382 | 0.00291612 | 0.000 |
| b10 CS - b5 PPMS | 0.01647102 | 0.00291612 | 0.000 |
| b10 CS - b6 PPMS | 0.01436646 | 0.00291612 | 0.000 |
| b10 CS - b7 PPMS | 0.01303365 | 0.00291612 | 0.003 |
| b10 CS - b8 PPMS | 0.01174572 | 0.00291612 | 0.018 |
| b10 CS - b2 RRMS | 0.01156537 | 0.00214992 | 0.000 |
| b10 CS - b4 RRMS | 0.00801681 | 0.00214992 | 0.050 |
| b10 CS - b5 RRMS | 0.00802014 | 0.00214992 | 0.050 |
| b2 PPMS - b5 PPMS | -0.00411651 | 0.00107445 | 0.032 |
| b2 PPMS - b6 PPMS | -0.00622107 | 0.00107445 | 0.000 |
| b2 PPMS - b7 PPMS | -0.00755388 | 0.00107445 | 0.000 |
| b2 PPMS - b8 PPMS | -0.00884181 | 0.00107445 | 0.000 |
| b2 PPMS - b9 PPMS | -0.01087193 | 0.00107445 | 0.000 |
| b2 PPMS - b10 PPMS | -0.01255273 | 0.00107445 | 0.000 |
| b2 PPMS - b3 RRMS | -0.01285556 | 0.00242502 | 0.000 |
| b2 PPMS - b4 RRMS | -0.01257073 | 0.00242502 | 0.000 |
| b2 PPMS - b5 RRMS | -0.01256739 | 0.00242502 | 0.000 |
| b2 PPMS - b6 RRMS | -0.01336562 | 0.00242502 | 0.000 |
| b2 PPMS - b7 RRMS | -0.01358712 | 0.00242502 | 0.000 |
| b2 PPMS - b8 RRMS | -0.01361114 | 0.00242502 | 0.000 |
| b2 PPMS - b9 RRMS | -0.01467055 | 0.00242502 | 0.000 |
| b2 PPMS - b10 RRMS | -0.01570709 | 0.00242502 | 0.000 |
| b3 PPMS - b6 PPMS | -0.00401227 | 0.00107445 | 0.045 |
| b3 PPMS - b7 PPMS | -0.00534508 | 0.00107445 | 0.000 |
| b3 PPMS - b8 PPMS | -0.00663302 | 0.00107445 | 0.000 |
| b3 PPMS - b9 PPMS | -0.00866314 | 0.00107445 | 0.000 |
| b3 PPMS - b10 PPMS | -0.01034393 | 0.00107445 | 0.000 |
| b3 PPMS - b3 RRMS | -0.01064676 | 0.00242502 | 0.004 |
| b3 PPMS - b4 RRMS | -0.01036193 | 0.00242502 | 0.007 |
| b3 PPMS - b5 RRMS | -0.01035859 | 0.00242502 | 0.007 |
| b3 PPMS - b6 RRMS | -0.01115682 | 0.00242502 | 0.002 |
| b3 PPMS - b7 RRMS | -0.01137832 | 0.00242502 | 0.001 |
| b3 PPMS - b8 RRMS | -0.01140234 | 0.00242502 | 0.001 |
| b3 PPMS - b9 RRMS | -0.01246176 | 0.00242502 | 0.000 |
| b3 PPMS - b10 RRMS | -0.0134983 | 0.00242502 | 0.000 |
| b4 PPMS - b7 PPMS | -0.00511017 | 0.00107445 | 0.001 |
| b4 PPMS - b8 PPMS | -0.0063981 | 0.00107445 | 0.000 |
| b4 PPMS - b9 PPMS | -0.00842822 | 0.00107445 | 0.000 |
| b4 PPMS - b10 PPMS | -0.01010902 | 0.00107445 | 0.000 |
| b4 PPMS - b3 RRMS | -0.01041185 | 0.00242502 | 0.007 |
| b4 PPMS - b4 RRMS | -0.01012701 | 0.00242502 | 0.010 |
| b4 PPMS - b5 RRMS | -0.01012368 | 0.00242502 | 0.010 |
| b4 PPMS - b6 RRMS | -0.01092191 | 0.00242502 | 0.003 |
| b4 PPMS - b7 RRMS | -0.01114341 | 0.00242502 | 0.002 |
| b4 PPMS - b8 RRMS | -0.01116743 | 0.00242502 | 0.002 |
| b4 PPMS - b9 RRMS | -0.01222684 | 0.00242502 | 0.000 |
| b4 PPMS - b10 RRMS | -0.01326338 | 0.00242502 | 0.000 |
| b5 PPMS - b8 PPMS | -0.00472531 | 0.00107445 | 0.003 |
| b5 PPMS - b9 PPMS | -0.00675542 | 0.00107445 | 0.000 |
| b5 PPMS - b10 PPMS | -0.00843622 | 0.00107445 | 0.000 |
| b5 PPMS - b6 RRMS | -0.00924911 | 0.00242502 | 0.038 |
| b5 PPMS - b7 RRMS | -0.00947061 | 0.00242502 | 0.028 |
| b5 PPMS - b8 RRMS | -0.00949463 | 0.00242502 | 0.027 |
| b5 PPMS - b9 RRMS | -0.01055404 | 0.00242502 | 0.005 |
| b5 PPMS - b10 RRMS | -0.01159059 | 0.00242502 | 0.001 |
| b6 PPMS - b9 PPMS | -0.00465086 | 0.00107445 | 0.005 |
| b6 PPMS - b10 PPMS | -0.00633166 | 0.00107445 | 0.000 |
| b6 PPMS - b10 RRMS | -0.00948603 | 0.00242502 | 0.027 |
| b7 PPMS - b10 PPMS | -0.00499885 | 0.00107445 | 0.001 |
| b2 RRMS - b3 RRMS | -0.0038334 | 0.00071563 | 0.000 |
| b2 RRMS - b4 RRMS | -0.00354856 | 0.00071563 | 0.000 |
| b2 RRMS - b5 RRMS | -0.00354523 | 0.00071563 | 0.000 |
| b2 RRMS - b6 RRMS | -0.00434346 | 0.00071563 | 0.000 |
| b2 RRMS - b7 RRMS | -0.00456495 | 0.00071563 | 0.000 |
| b2 RRMS - b8 RRMS | -0.00458898 | 0.00071563 | 0.000 |
| b2 RRMS - b9 RRMS | -0.00564839 | 0.00071563 | 0.000 |
| b2 RRMS - b10 RRMS | -0.00668493 | 0.00071563 | 0.000 |
| b3 RRMS - b10 RRMS | -0.00285153 | 0.00071563 | 0.018 |
| b4 RRMS - b10 RRMS | -0.00313637 | 0.00071563 | 0.004 |
| b5 RRMS - b10 RRMS | -0.00313971 | 0.00071563 | 0.004 |
